# Benzodiazepine-induced anterograde amnesia: detrimental side effect to novel study tool

**DOI:** 10.3389/fphar.2023.1257030

**Published:** 2023-09-14

**Authors:** Kameron Kaplan, Holly Christian Hunsberger

**Affiliations:** ^1^ Center for Neurodegenerative Diseases and Therapeutics, Rosalind Franklin University of Medicine and Science, North Chicago, IL, United States; ^2^ Department of Neuroscience, Rosalind Franklin University of Medicine and Science, The Chicago Medical School, North Chicago, IL, United States

**Keywords:** benzodiazepines, anterograde amnesia, sex differences, Alzheimer’s disease, GABAA, fear conditioning

## Abstract

Benzodiazepines (BZDs) are anxiolytic drugs that act on GABAa receptors and are used to treat anxiety disorders. However, these drugs come with the detrimental side effect of anterograde amnesia, or the inability to form new memories. In this review we discuss, behavioral paradigms, sex differences and hormonal influences affecting BZD-induced amnesia, molecular manipulations, including the knockout of GABAa receptor subunits, and regional studies utilizing lesion and microinjection techniques targeted to the hippocampus and amygdala. Additionally, the relationship between BZD use and cognitive decline related to Alzheimer’s disease is addressed, as there is a lack of consensus on whether these drugs are involved in inducing or accelerating pathological cognitive deficits. This review aims to inspire new research directions, as there is a gap in knowledge in understanding the cellular and molecular mechanisms behind BZD-induced amnesia. Understanding these mechanisms will allow for the development of alternative treatments and potentially allow BZDs to be used as a novel tool to study Alzheimer’s disease.

## 1 Introduction

Benzodiazepines (BZDs), a class of anxiolytic drugs, were invented in the mid-1950s by chemist Leo Sternbach. In an effort to create a perfect tranquilizer, Sternbach and his associate Earl Reeder stumbled upon the compound that would soon become the first clinically available BZD. The drug chlordiazepoxide, also known as Librium, was introduced in the 1960s and revolutionized the clinical use of sedatives (Sternbach, 1979). Subsequently, other BZDs like diazepam were released in the early 1960s to improve the efficacy and potency of this new class of drugs (Sternbach, 1979). However, these drugs came with side effects, including impaired motor coordination, vertigo, mood swings, and anterograde amnesia (Griffin et al., 2013).

Although anterograde amnesia is desirable during perioperative surgical periods and times of heightened anxiety, the long-lasting cognitive fog reported by patients needs to be addressed and better understood. Anterograde amnesia, the inability to form new memories, was first observed in 1972 after intravenous injections of 10 mg of diazepam resulted in a reduction of recognition memory in 90% of women (Dundee and Pandit, 1972). This amnesia had a rapid onset of peak incidence 2–3 min after injection, with effects lasting around 1 h (Dundee and Pandit, 1972). More recently, healthy individuals showed impaired recollection of stories encoded immediately after diazepam administration (Segura et al., 2021). Interestingly, this amnesia is similar to the amnesia in patients with severe medial temporal damage (Segura et al., 2021). Acknowledging this observation, studying the relationship between BZD use in aging and Alzheimer’s disease (AD) is of great interest, as AD also results in atrophy of the medial temporal lobe. The cellular and molecular mechanisms underlying BZD-induced anterograde amnesia and cognitive impairment are not fully understood. Understanding these mechanisms could give greater insight into how these drugs might contribute to cognitive decline in aging and AD. This review outlines the historical and current literature surrounding BZDs and anterograde amnesia, intending to inspire new research directions.

## 2 Behavioral studies to measure anterograde amnesia

Scientists turned to rodent models to understand the cellular and molecular mechanisms underlying BZD-induced anterograde amnesia. Memory tasks used to study the amnestic properties of BZDs include the 1) passive avoidance task (PAT), 2) elevated plus maze (EPM), and 3) contextual fear conditioning (CFC). Early studies using the PAT showed that BZDs induce anterograde amnesia in mice, as mice that received lorazepam before training entered an aversive context significantly quicker than controls during the re-exposure test (Table 1) (E. R. Gamzu, 1988). This result was later confirmed in rats in a dose-dependent manner (Pain et al., 2002). Rats receiving high doses of midazolam before training in a PAT showed decreased latency to enter an aversive context compared to controls during re-exposure, indicating the inability to remember the initial aversive experience (Pain et al., 2002).

**TABLE 1:** Amnestic effects of benzodiazepines. Table 1 summarizes changes in memory from all BZD studies throughout the review.

Human	Article	Drug	Sex	Behavior	Route	Memory	Result
Drug before training	Dundee and Pandit (1972)	Diazepam	W	Recognition memory	IV	Impairment	Recognition memory was impaired in women after diazepam administration
	Segura et al. (2021)	Diazepam	N/A	Recollection	Oral		Impaired recollection of stories encoded after diazepam administration
	Pomara et al. (2005)	Lorazepam	M/W	Verbal Recall 5 h later	Oral		In APOE4 carriers show persistent deficits in long term memory compared to APOE3 carriers after Lorazepam administration
	Stonnington et al. (2017)	Lorazepam	M/W	Working memory, visuospatial memory, and executive function	Oral		APOE3/4 carriers show deficits 2.5 and 5 h after benzo administration
Rodent							
Drug before training	Gamzu (1988)	Lorazepam	N/A	PAT	Oral	Impairment	Treated mice show decreased latency to aversive context
	Pain et al. (2002)	Midazolam	M	PAT	ip		Treated rats show decreased latency to an aversive context
	Malkani and Rosen (2000)	Diazepam	M	CFC	ip		Treated mice show significantly less freezing during re-exposure test
	Wan et al. (2004)	Lorazepam	M	NOR	Intra perirhinal cortex/ip		Treated mice show impaired recognition memory
	Vandesquille et al. (2012)	Alprazolam	M	4-hole board apparatus	ip		Memory recovered after use of Alzheimer’s drugs
	Timic et al. (2013)	Midazolam	M	MWM	ip		Impaired spatial but not procedural memory
	Murphy et al. (2022)	Midazolam	F	MWM	Oral		No spatial memory impairment, but hypothesized procedural memory impairment
	Gibbs et al. (1998)	Lorazepam	F	PAT	ip		Amnesia attenuated after estrogen replacement
	Silva et al. (2016)	Diazepam	M/F	PM-DAT	ip		Diazepam induced amnesia found to be prevented in proestrus females
	Singh et al. (1998)	Alprazolam	M/F	EPM	ip		The inhibitor (flumazenil) significantly reduced the anterograde and retrograde amnesia induced by benzodiazepines
	Rudolph et al. (1999)	Diazepam	N/A	PAT	Oral		a1 subunit deficient mice show comparable latency to the aversive context compared to controls
	Giachero et al. (2015)	Midazolam	M	CFC	Intra-BLA injection		Intra-BLA injection of midazolam reduces fear expression
	Tomaz et al. (1991)	Diazepam	M	Continuous multiple-trial inhibitory avoidance test	ip		Amygdala Complex lesions prevent retention deficits induced by benzodiazepines
	Tomaz et al. (1992)	Diazepam	M	IAT	ip/microinjections to amygdala nuclei		Retention deficits present in rats with central and lateral amygdala nuclei. Retention deficits were not present in rats with BLA lesions
	Whittington et al. (2019)	Midazolam	M	BM	ip		No deterioration of spatial memory in hTau mice after treatment
	Liu et al. (2022)	Remimazolam Tosylate	F	NOR/OPR	ip		Short-term cognitive decline/Protected memory 1 month later
Drug 5 min after training	Bustos et al. (2006)	Midazolam	M	CFC	ip		Amnesia is induced when administered immediately after the reconsolidation period. Not immediately after training
	Gafford et al. (2005)	Midazolam	M	Auditory/Contextual Fear Conditioning	Intrahippocampal		Amnesia is induced immediately, but not 3 h after training
Drug 5 min after re-exposure	Bustos et al. (2006)	Midazolam	M	CFC	ip		Amnesia induced after Midazolam was administered immediately after a re-exposure period
Drug before training and re-exposure	Nakagawa et al. (1993)	Diazepam	M	PAT	ip	State dependent	State-dependent memory was observed after administration of diazepam
	Jovasevic et al. (2015)	Gaboxadol	M	CFC	Intrahippocampal	State dependent	Contextual fear memory was state dependent with administration of Gaboxadol
	Sanday et al. (2012)	Midazolam	M	PM-DAT	ip	State dependent	Discriminative memory was not state dependent. Non-associative habituation memory may be state dependent
	Gazmu (1988)	Lorazepam	N/A	PAT	Oral	No Impairment	There was no state-dependent memory observed in the PAT after lorazepam administration

W, women; M, men; IV, intravenous; ip, intraperitoneal; M, male; F, female; PAT, passive avoidance task; CFC, contextual fear conditioning; NOR, novel object recognition; OPR, object place recognition; MWM, morris water maze; PM-DAT, plus maze-discriminative avoidance task; EPM, elevated plus maze; BM, barnes maze; IAT, inhibitory avoidance task.

Similar results are observed when diazepam is injected prior to training in a 1-shock aversive CFC paradigm. Freezing behavior during context re-exposure is measured as a proxy for memory. Treated rats freeze significantly less during CFC re-exposure than controls (Malkani and Rosen, 2000). However, different timelines of BZD administration have yielded different results. Midazolam injected 5 min after CFC does not decrease freezing levels during re-exposure (Bustos et al., 2006), but midazolam injected 5 min after the re-exposure session decreases freezing levels in a subsequent test period 24 h later. These results suggest that midazolam impairs the reconsolidation of a memory rather than the initial consolidation phase (Bustos et al., 2006). However, many drugs can also induce state-dependent memory, which further complicates our analysis of memory impairments (Zarrindast and Khakpai, 2020).

### 2.1 State-dependent memory

State-dependent memory is the retrieval of memory more easily facilitated when the organism is in a similar physiological state to when the memory was first encoded. For example, injection of diazepam induced state-dependent learning in the PAT when given 30 min before training and 30 min before re-exposure, as latency to the aversive chamber was similar to controls in this timeline (Table 1) (Nakagawa et al., 1993). Similarly, intra-hippocampal injections of gamma amino butyric acid (GABA) a agonist, gaboxadol, induced state-dependent learning in a CFC paradigm (Jovasevic et al., 2015).

However, other studies have found that state-dependent memory is not always observed in PAT and Plus Maze-Discriminative Avoidance Task (PM-DAT) paradigms. Contrary to the previous studies, mice receiving oral administration of lorazepam (L) pre-training, regardless of pre-test treatment, entered the aversive context in significantly less time than mice receiving a vehicle (V) before training (V-L or V-V) (E. R. Gamzu, 1988). Similarly, in the PM-DAT, in which one enclosed arm contains aversive stimuli, including a bright light and a loud sound-generating machine, mice pre-treated with both saline and midazolam before training sessions acquired proficiency in the discriminative avoidance task. Upon testing, midazolam-treated groups exhibited a learning impairment as groups receiving saline pre-training (Sal-Sal or Sal-MDZ) spent significantly more time in the non-aversive arms, while groups receiving midazolam pre-training (MDZ-Sal or MDZ-MDZ) did not exhibit significant differences in time spent between the aversive and the non-aversive arms (Sanday et al., 2012). The failure to discriminate between the aversive and the non-aversive areas when midazolam was administered pre-training and pre-test indicates that state-dependent memory was not utilized in this discriminative task.

The differences in results between studies may be attributed to the different drugs used in each study. Different BZDs have various time courses of action and require different dosages to achieve desired effects. Standardizing experiments to use the same drug may result in more precise results. A summary of all behavioral studies and timelines is included in Table 1. To truly understand the relationship between BZDs and state-dependent learning, using other memory tasks could lead to more answers. Examples include consolidation-based memory tests and altering the timelines from training to the test.

### 2.2 Recognition and spatial memory impairments

Although prior amnestic studies mainly used aversive contextual or avoidance learning, recognition and spatial memory tests may provide clues to determine whether BZDs are truly detrimental to cognition. For example, male rats injected with lorazepam before a novel object acquisition phase exhibited impaired recognition memory during the test day (Table 1) (Wan et al., 2004). This effect was also present when lorazepam was administered locally into the perirhinal cortex via cannula injection (Wan et al., 2004). Drug-induced amnesia is also exhibited in delayed spatial learning and discrimination tasks, including the 4-hole board apparatus where mice search for food. A small dose of 0.1 mg/kg of alprazolam given before the acquisition phase induced amnesia in mice, as alprazolam-treated mice exhibited significantly fewer nose pokes in the previously baited hole than controls (Vandesquille et al., 2012). Surprisingly, mice treated with both alprazolam and AMPA receptor-positive allosteric modulators together did not exhibit BZD-induced amnesia (Vandesquille et al., 2012). These data support the hypothesis that increased GABAergic inhibitory neurotransmission mediates drug-induced amnesic effects. Spatial memory assessed using the Morris Water Maze is also impaired. Specifically, male rats pre-treated with midazolam show impaired acquisition and retention of spatial learning memory but not procedural memory (Timić et al., 2013). A more recent study found the opposite effect in which female rats, pre-treated with midazolam, showed no impairment of spatial working memory but were hypothesized to have impaired procedural memory (Murphy et al., 2022). These contradictory observations may be due to sex differences or opposite circadian cycles between studies (Murphy et al., 2022).

### 2.3 Sex differences in benzodiazepine research

Although sex differences in drug-related research have been largely ignored, scientists have begun to examine how hormones influence the effects of BZDs. For example, ovariectomized female rats treated with estrogen replacement show attenuation of lorazepam-induced amnesia in the PAT during re-exposure (Gibbs et al., 1998). This suggests that estrogen has a protective effect that can reverse drug-induced amnesia in female rodents. Serum levels of lorazepam in both estrogen-treated and non-estrogen-treated rats were similar, indicating that the attenuation of lorazepam-induced amnesia was not caused by unbalanced drug levels in the blood, further confirming that estrogen is somehow responsible for this effect (Gibbs et al., 1998). More recent studies looking directly at sex differences show that high doses of diazepam (2 mg/kg and 4 mg/kg) impair memory retrieval in both sexes in a PM-DAT (Silva et al., 2016). Upon further examination, results indicate that the estrous cycle phase also influences the amnesic effect in females. Female rats in metestrus, diestrus, and estrus phases showed a significant amnesic effect when injected with 2 and 4 mg/kg of diazepam. Contrarily, female rats in proestrus did not exhibit amnesia at either 1 or 2 mg/kg doses (Silva et al., 2016). Finally, pre-treatment in proestrus female rats with a 5-alpha reductase inhibitor (finasteride) or a progesterone antagonist (mifepristone) restored the amnesic effects induced by 2 mg/kg doses of diazepam (Silva et al., 2016). These results point to sex hormones as mediators that influence BZD-induced amnesia. With limited research analyzing sex differences in this subject, further research should determine whether hormones affect drug-induced amnesia in males, how this changes with age and menopause models, and how this impacts neuronal activity in different brain regions.

It was and is clear that BZD-induced amnesia can be studied in rodents, but the exact injection timeline depends on the scientific questions being asked. Still, many unanswered questions remained- How would chronic administration impact cognition, does age or sex play a role, what brain regions are involved, and could these impairments be reversed by targeting BZD sites?

## 3 Molecular mechanisms of anterograde amnesia

Because BZDs are classified as positive allosteric modulators of the gamma amino butyric acid (GABA)-a receptor (Griffin et al., 2013), GABA receptor antagonists were used in an attempt to reverse BZD-induced amnesia. While GABA antagonists were ineffective, the BZD binding site competitive antagonist, Ro 15-1788 (Flumazenil), significantly reversed amnesia in the PAT model (E. R. Gamzu, 1988). Additionally, flumazenil significantly reduced the anterograde and retrograde amnesia produced by alprazolam in the EPM, in which transfer latency to a novel region of the maze from day 1–2 was used as a measure of memory (Singh et al., 1998). These early findings suggest that BZD binding sites may play a role in the amnesic effects. However, as GABAa receptors express a variety of other ligand-binding sites specific to ligands such as barbiturates, toxins, and other anesthetics (Sigel and Ernst, 2018), further analysis of how these receptors respond to different ligands may give better insight into how BZDs produce amnesia.

### 3.1 GABA receptors

**FIGURE 1:**
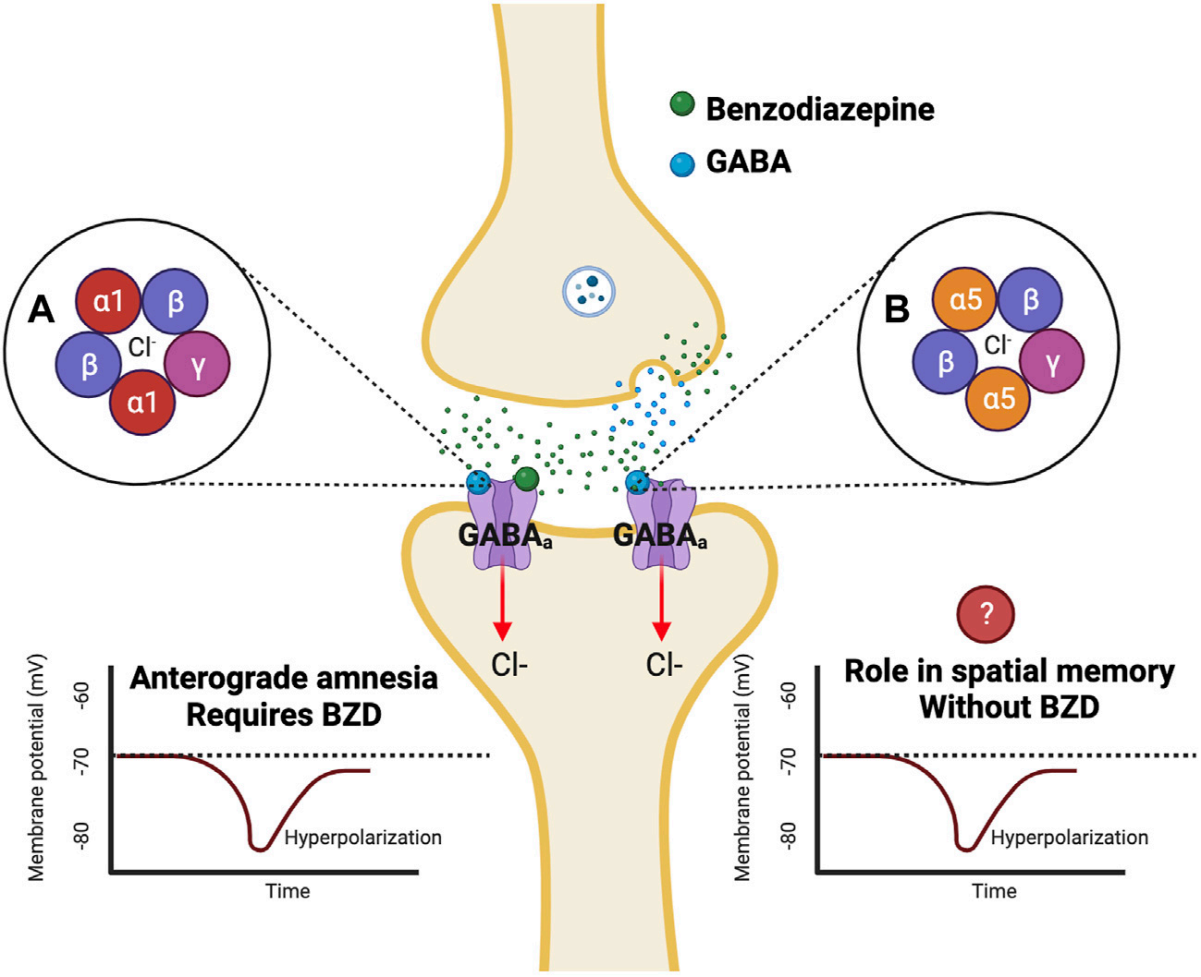
Benzodiazepines affect varying isoforms of GABAa receptors which are potentially responsible for anterograde amnesia. Benzodiazepines are positive allosteric modulators that bind to GABAa receptors at a BZD site separate from the GABA binding site. Binding of a benzodiazepine to the GABAa receptor results in an influx of Cl-into the cell leading to hyperpolarization (Griffin et al., 2013). Previous studies have found that α1 knockout mutant mice do not exhibit BZD-induced anterograde amnesia in the PAT test (Rudolph et al., 1999). Additional studies have indicated a role for the α5 subunit in learning and memory in fear conditioning and spatial memory tests (Collinson et al., 2002; Crestani et al., 2002).

GABAa receptors can be categorized as ligand-gated ion channels, permeable to both chloride and bicarbonate anions (Sigel and Ernst, 2018). BZDs bind to separate BZD sites on GABAa receptors, which leads to an increase in both GABA-activated channel openings and ion channel conductance of chloride (Figure 1) (Chebib and Johnston, 2000). This leads to hyperpolarization and reduced excitability of neurons (Griffin et al., 2013). GABAa receptors are comprised of subunits arranged in a pentameric circular structure (Sigel and Ernst, 2018). These receptors can exhibit many different isoforms from a combination of 19 subunits, including alpha (α1-6), beta (β1-3), gamma (γ1-3), ბ, ε, π, θ, and ρ1-3 (Engin et al., 2018). The most expressed GABAa receptor isoform is comprised of two α1, two β2, and one γ2 subunit (α1β2γ2) and accounts for around 60% of all GABAa receptors in the brain (Engin et al., 2018). The BZD binding site is located between the interface of the α and γ subunit and can be characterized as diazepam-sensitive (DS) or diazepam-insensitive (DI) based on the α isoform present within the receptor (Sigel and Ernst, 2018). For example, GABAa receptors containing α1,2,3 & 5 and γ1-3 are considered (DS), while others containing the α4 and 6 subunits are considered (DI) (Sigel and Ernst, 2018). The alpha (α) subunit is of interest, as it is thought to mediate the anxiolytic, sedative, relaxant, and perhaps the amnesic effects of BZDs (Griffin et al., 2013). It will be essential to understand how BZDs affect different GABAa receptor subtypes, as different isoforms of the GABAa receptor have specific subcellular expression patterns, which lead to varying channel-gating actions and unique pharmacological properties (Engin et al., 2018). While GABAa receptors containing α2 and α3 subunits are concentrated more in synaptic regions, α4 and α5 subunits are located more in perisynaptic and extrasynaptic areas (Engin et al., 2018). The localization of GABAa receptors to these areas is specific for the mechanism of inhibition being utilized, as extrasynaptic receptors allow for tonic inhibition, while synaptic receptors mediate phasic inhibition (Engin et al., 2018). Therefore, analysis of the α subunits contributing to BZD-induced amnesia will allow for a better understanding of the mechanism of inhibition that is altered. Most synaptic GABAa receptors are anchored to the inhibitory synapse by Gephyrin scaffolding proteins based on their subunit composition, while others can diffuse laterally through the plasma membrane and be recruited to the synapse (Jacob et al., 2008). Different scaffolding proteins like Radixin anchor extrasynaptic receptors containing the α5 subunit (Jacob et al., 2008). However, these receptors can be recruited to the synapse during times of increased excitation (Hausrat et al., 2015). Both the α1 and α5 subunits are highly expressed in the hippocampus (Hörtnagl et al., 2013), with α5 subunits being largely expressed in hippocampal pyramidal cells (Möhler and Rudolph, 2017). These two subunits are thought to play a role in amnesic side effects, learning, and memory; therefore, they have been heavily researched regarding their role in BZD-induced amnesia.

To understand the importance of the α1 subunit of GABAa receptors, a point mutation was inserted into a mouse line that replaced a histidine with arginine in the α1 subunit gene, making it insensitive to BZDs (Rudolph et al., 1999). When put through a PAT with diazepam administered 30 min before training, mutant mice showed comparable latency to aversive stimuli compared to controls during recall, meaning they did not experience BZD-induced amnesia, suggesting that the α1 subunit is responsible for the amnestic effects (Rudolph et al., 1999). Comparably, this same mutation was introduced into the α5 subunit gene. Without a BZD injection, freezing levels in CFC and delayed fear conditioning in mutant mice were similar to controls (Crestani et al., 2002). However, mutant mice exhibited a significant increase in freezing during a trace fear conditioning test, indicating that this subunit alone plays a role in learning and memory (Crestani et al., 2002). Additional studies using α5 subunit knockout globally and specific to CA1 cells found similar and contradictory results. Global and CA1 specific α5 knockdown mice show a significant increase in freezing during trace fear conditioning, similar to previous studies (Engin et al., 2020). In contrast to previous findings, mice with global α5 knockdown but not CA1-specific knockdown showed significantly increased freezing in a CFC paradigm (Engin et al., 2020). Acknowledging this result, future studies should analyze if BZD-induced amnesia is present in these α5 knockout mice using CFC.

Recent studies have used the positive allosteric modulator of GABAa receptors, etomidate, known for its memory impairment properties, to understand how α5 subunits on both pyramidal and interneurons affect memory in a CFC paradigm (Zhu et al., 2023). Results indicate that etomidate affects α5 subunits in pyramidal and interneurons to impair memory, as mice with α5 knockouts in both cell types exhibited comparable freezing with and without etomidate treatment, while wild-type controls exhibited significantly less freezing after etomidate treatment (Zhu et al., 2023). While etomidate and BZDs both have memory impairment effects, these compounds have different binding sites on GABAa receptors (Chiara et al., 2013). This further supports the need to conduct additional research using BZDs to see if they impair memory using a similar mechanism. The α5 subunit may also be pertinent for spatial memory, as mice with an α5 knockout mutation perform significantly better in the “matching to place” version of the Morris water maze (Collinson et al., 2002). Interestingly, intrahippocampal knockdown of the α5 subunit using antisense oligonucleotides did not impair auditory contextual fear memory when midazolam was given directly after training (Gafford et al., 2005). While results point to the α1 subunit as a possible mediator for BZD-induced amnesia, the role of the α5 subunit is still ambiguous. Knockout of the α5 subunit has been shown to affect memory; however, these mutations did not affect BZD-induced amnesia. Administration of BZDs in an α5 knockout mutant before the encoding period may further delineate the α5 subunit’s role in memory formation. Further research must decipher how the α5 subunit modulates learning and memory.

### 3.2 Brain regions involved in anterograde amnesia

Although there have been proposed mechanisms by which BZDs induce anterograde amnesia, no single brain region has been identified as responsible for this effect. With the knowledge that the hippocampus and the amygdala are important regions for fear memory, scientists chose to focus on these areas first. Intrahippocampal injections of midazolam targeting the dorsal hippocampus impairs contextual fear memory when given immediately after training (Gafford et al., 2005). However, the same treatment does not impair fear memory when given 3 hours after the training period (Gafford et al., 2005) or given i.p. immediately after training (Bustos et al., 2006). These results suggest that BZDs impair the ability of the hippocampus to consolidate new memories only after intrahippocampal injection. Future research should focus on the administration route of BZDs. Integration of BZDs into the diet of rodents could mimic the oral administration usually used by humans. Additional research should focus on the relationship between BZD injection and spine density, neuronal projections, and immediate early genes in different hippocampal regions as spine densities decrease in the CA1 and CA3 following chronic diazepam use in young and old mice (Furukawa et al., 2021).

The amygdala processes emotion and has a strong connection with the hippocampus (Maren, 2001). Therefore, the hippocampal-amygdala pathway is worthy of attention to try to understand how BZDs induce amnesia for fear memories. Intra-basolateral amygdala injections of midazolam before stress exposure prevents CA1 structural plasticity and reduces fear expression (Giachero et al., 2015). Previous studies have also deemed the amygdala complex (AC) important for fear memory. Both control and high-dose diazepam-treated rats with bilateral amygdaloid complex lesions showed impaired acquisition in a continuous multiple-trial inhibitory avoidance test (Tomaz et al., 1991). When re-exposed 48 h later, diazepam-treated rats with AC lesions did not show a significant difference in latency to the aversive context compared to controls, indicating that the amygdaloid complex may be crucial for the amnesic effects of BZDs (Tomaz et al., 1991). To further understand which amygdala nuclei were relevant for this amnesia, the central, lateral, and basolateral amygdala were lesioned. Rats with lesions in the central and lateral amygdala exhibited impaired retention after diazepam treatment; however, rats with lesions in the basolateral amygdala did not exhibit retention deficits (Tomaz et al., 1992). Finally, microinjections of diazepam into the basolateral and lateral nuclei of the amygdala induced amnesia in rats (Tomaz et al., 1992). Together, these findings suggest that the basolateral nucleus of the amygdala is crucial for BZD-induced amnesia; however, the affected hippocampal regions are still largely unidentified. Although lesions in the central amygdala did not lead to impaired retention in previous studies, this brain region should still be analyzed. The immediate early gene c-fos, indicative of recent activity, is increased in PKCδ+ neurons in the central amygdala after BZD administration (Griessner et al., 2021). This increase in c-fos has also been correlated with the anxiolytic activity exhibited by mice in the EPM (Griessner et al., 2021). Analysis of the neuronal circuits that include the central amygdala may give further insight into the amnesic effects of BZDs. As previously stated, BZD-induced amnesia shows similarities to the amnesia present in patients with severe damage to the medial temporal lobe, a hallmark of AD. Acknowledging this similarity, research should analyze the relationship between BZDs, aging, and AD.

## 4 Benzodiazepines and Alzheimer’s disease

### 4.1 Human studies

As of 2008, 5.2% of the US population aged 18 to 80 reported using BZDs (Olfson et al., 2015). Of the 5.2%, women reported using BZDs almost twice as much as men. Older populations generally report more long-term use than younger populations (Olfson et al., 2015), and BZDs increase adverse drug reactions in elderly patients (Larson et al., 1987). Therefore, understanding how BZD use affects AD patients is needed, as this disease is more prevalent in older populations and women. Studies related to BZDs and how they affect memory in AD patients date back to the late 1980s (Sunderland et al., 1989). While early studies failed to reveal memory deficits between AD patients and age-matched controls, they did indicate a high rate of adverse drug reactions. A population of elderly patients experiencing adverse drug reactions was found to have cognitive impairments associated with long-acting BZD use (Larson et al., 1987). The effects of these drugs on cognitive decline in AD patients have been further analyzed using models such as the Mini-Mental State Exam (MMSE) and the Clinical Dementia Rating Sum of Boxes (CDR-Sum) (Rosenberg et al., 2012; Borda et al., 2021). Constant use of BZDs has been associated with a rapid decline in MMSE and a rapid increase in CDR-Sum (Rosenberg et al., 2012). However, controversy still exists as recent work failed to show an association between BZD use and accelerated cognitive decline in AD patients and elderly patients with normal cognition using these same measures (Zhang et al., 2016; Borda et al., 2021). The lack of consensus on whether BZDs are associated with cognitive decline could be because of different confounding factors within the studied populations. These include the consumption of other drugs and the onset of BZD usage. Controlled longitudinal studies could try to limit these confounding factors to reach a more precise answer.

Studies focusing on the APOE4 gene, an AD risk factor, show deficits in long-term memory and cognitive function after BZD administration (Pomara et al., 2005; Stonnington et al., 2017). Certain patients with the APOE4 gene exhibit persistent deficits in long-term memory compared to controls after lorazepam administration (Pomara et al., 2005). Compared to people with the APOE 3/3 gene, carriers of the APOE 3/4 gene show significantly more cognitive impairments related to working memory, visuospatial memory, and executive function 2.5 and 5 h after administration of lorazepam (Stonnington et al., 2017). These studies suggest that AD risk factor gene carriers are more susceptible to cognitive impairments induced by BZDs. There is no current consensus on whether BZDs increase the risk of developing AD or whether these drugs accelerate cognitive decline in previously diagnosed patients. Future research should look to see if BZDs aggravate AD pathology.

### 4.2 Rodent studies

Although BZD studies are contradictory in the patient population, we can use AD mouse models to begin answering these complicated questions. Tau phosphorylation, a hallmark of AD, is increased 30 min and 6 h later in wild-type mice injected with midazolam (Whittington et al., 2019). Surprisingly, these same injections did not cause further tau phosphorylation in transgenic hTau mice and did not impair spatial reference memory (Whittington et al., 2019). These results suggest that BZDs may not accelerate pathology but may induce disease hallmarks. Comparably, the injection of a new BZD, remimazolam tosylate, increased tau phosphorylation in certain areas of the frontal cortex in the short term but actually reduced tau phosphorylation in frontal cortex areas over time (Liu et al., 2022). Recognition memory was also impaired short term, but these deficits did not last (Liu et al., 2022). This is similar to other findings indicating that these drugs impair working memory during treatment but do not persist after treatment discontinuation (Carton et al., 2021). Perhaps one of the more interesting findings is that the amnesia produced by BZDs may be similar to the memory deficits observed in AD patients, as Alzheimer’s drugs such as memantine (an NMDA receptor antagonist) and donepezil (an acetylcholinesterase inhibitor) can reverse alprazolam-induced amnesia (Vandesquille et al., 2012). Based on these findings, BZDs could also be used as a novel tool to help understand memory and cognitive impairments produced by AD.

## 5 Future directions in benzodiazepine research

**FIGURE 2:**
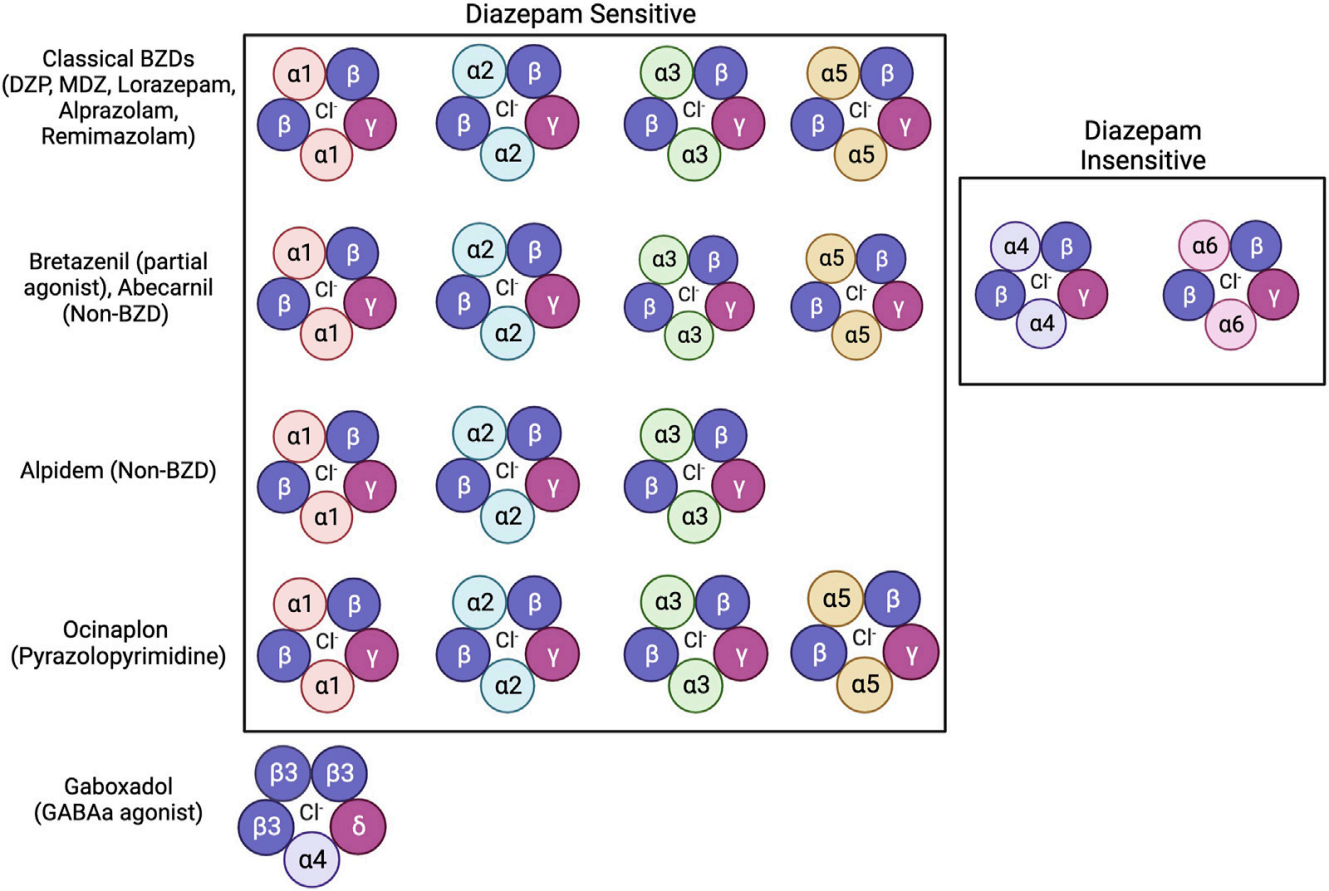
Benzodiazepines, non-benzodiazepines, and other anxioselective compounds show affinity for specific isoforms of GABAa receptors. Classical BZDs have high affinity to DS (Diazepam Sensitive) isoforms of GABAa receptors which include α1, α2, α3, and α5 subunits (Sigel and Ernst, 2018). GABAa receptor isoforms containing α4 and α6 subunits are categorized as DI (Diazepam Insensitive) (Sigel and Ernst, 2018). The anxioselective compounds Bretazenil (partial agonist), and Abecarnil (Non-BZD) have affinity to both DS and DI GABAa receptors (Mehta and Shank, 1995; Pym et al., 2005). The anxioselective drug Alpidem (Non-BZD) has affinity to α1, α2, and α3 isoforms, but not the α5 isoform (Sieghart and Savic, 2018). Another anxioselective compound, Ocinaplon (pyrazolopyrimidine) has affinity to all DS isoforms, with higher affinity to α1 compared to α5 (Lippa et al., 2005). The GABAa agonist gaboxadol is known to bind to GABAa receptors containing the α4 and ბ subunits (Zanettini et al., 2016; Sente et al., 2022).

Even with extensive research exploring how BZDs impair memory, many questions remain unanswered on the behavioral, molecular, and circuit levels. While many studies suggest that BZD binding sites mediates BZD-induced amnesia, other anxiolytic drugs that bind to the BZD binding site do not produce this amnesia (E. R. Gamzu, 1988). These include “anxioselective” drugs such as bretazenil, abecarnil, alpidem, and ocinaplon, which exhibit anxiolytic properties without the side effects of traditional BZDs, including amnesia (Skolnick, 2012). These compounds range in their affinity and selectivity for varying isoforms of GABAa receptors, with some acting as full agonists to receptors containing DS binding sites while others act as partial agonists on receptors with DS and DI binding sites (Figure 2) (Mehta and Shank, 1995; Lippa et al., 2005; Pym et al., 2005; Sieghart and Savic, 2018). Understanding how these anxioselective drugs affect GABAa receptors differently compared to classical BZDs, newer BZD compounds like remimazolam, and other GABAa agonists like gaboxadol could point to new directions regarding how BZDs induce amnesia through different isoforms (Zanettini et al., 2016; Noor et al., 2021; Sente et al., 2022). Evidence strongly suggests that the α1 and α5 subunits of GABAa receptors are also mediators of drug-induced amnesia for different types of memory. New research should look to differentiate which memories are influenced by these subunits. As distinct pharmacological compounds that activate the GABAa receptor may induce different cascades, investigating secondary signaling pathways is essential for future studies. Because sex hormones, such as estrogen and progesterone, protect against drug-induced amnesia, the molecular mechanisms regarding sex differences must also be addressed. Additionally, results have indicated that the BLA of the amygdala mediates anterograde amnesia in BZD-treated rats, but the role of the hippocampus is still unclear. Viral tracing studies and activity-dependent memory tagging mouse lines may give further insight into how these two areas communicate. Finally, the relationship between BZDs and AD is still perplexing. Studies show that BZDs can induce hallmarks of Alzheimer’s but that drug-induced cognitive impairments that mirror AD eventually fade. Therefore, in addition to identifying the mechanism of action behind BZD-induced amnesia, future studies can use BZDs as a novel tool to study AD.
